# Tracking neuroinflammatory biomarkers in Alzheimer’s disease: a strategy for individualized therapeutic approaches?

**DOI:** 10.1186/s12974-024-03163-y

**Published:** 2024-07-30

**Authors:** Simone Lista, Bruno P. Imbimbo, Margherita Grasso, Annamaria Fidilio, Enzo Emanuele, Piercarlo Minoretti, Susana López-Ortiz, Juan Martín-Hernández, Audrey Gabelle, Giuseppe Caruso, Marco Malaguti, Daniela Melchiorri, Alejandro Santos-Lozano, Camillo Imbimbo, Michael T. Heneka, Filippo Caraci

**Affiliations:** 1 i+HeALTH Strategic Research Group, Department of Health Sciences, Miguel de Cervantes European University (UEMC), 47012 Valladolid, Spain; 2 Department of Research and Development, Chiesi Farmaceutici, 43122 Parma, Italy; 3 Oasi Research Institute-IRCCS, 94018 Troina, Italy; 4 2E Science, 27038 Robbio, Italy; 5 Studio Minoretti, 23848 Oggiono, LC Italy; 6 CMRR, Memory Resources and Research Center, Montpellier University of Excellence i-site, 34295 Montpellier, France; 7 Department of Drug and Health Sciences, University of Catania, 95125 Catania, Italy; 8 Department for Life Quality Studies, Alma Mater Studiorum, University of Bologna, 40126 Bologna, Italy; 9 Department of Physiology and Pharmacology, Sapienza University, 00185 Rome, Italy; 10 Physical Activity and Health Research Group (PaHerg), Research Institute of the Hospital, 12 de Octubre (‘imas12’), 28041 Madrid, Spain; 11 Department of Brain and Behavioral Sciences, University of Pavia, 27100 Pavia, Italy; 12 Luxembourg Centre for Systems Biomedicine, University of Luxembourg, 4367 Esch-Belval, Luxembourg

**Keywords:** Alzheimer’s disease, Neuroinflammation, Biomarkers, GFAP, YKL-40, ATI(N) classification system, Microglia, Astrocytes, Longitudinal studies, Clinical trials

## Abstract

**Background:**

Recent trials of anti-amyloid-β (Aβ) monoclonal antibodies, including lecanemab and donanemab, in early Alzheimer disease (AD) showed that these drugs have limited clinical benefits and their use comes with a significant risk of serious adverse events. Thus, it seems crucial to explore complementary therapeutic approaches. Genome-wide association studies identified robust associations between AD and several AD risk genes related to immune response, including but not restricted to *CD33* and *TREM2*. Here, we critically reviewed the current knowledge on candidate neuroinflammatory biomarkers and their role in characterizing the pathophysiology of AD.

**Main body:**

Neuroinflammation is recognized to be a crucial and contributing component of AD pathogenesis. The fact that neuroinflammation is most likely present from earliest pre-stages of AD and co-occurs with the deposition of Aβ reinforces the need to precisely define the sequence and nature of neuroinflammatory events. Numerous clinical trials involving anti-inflammatory drugs previously yielded unfavorable outcomes in early and mild-to-moderate AD. Although the reasons behind these failures remain unclear, these may include the time and the target selected for intervention. Indeed, in our review, we observed a stage-dependent neuroinflammatory process in the AD brain. While the initial activation of glial cells counteracts early brain Aβ deposition, the downregulation in the functional state of microglia occurs at more advanced disease stages. To address this issue, personalized neuroinflammatory modulation therapy is required. The emergence of reliable blood-based neuroinflammatory biomarkers, particularly glial fibrillary acidic protein, a marker of reactive astrocytes, may facilitate the classification of AD patients based on the ATI(N) biomarker framework. This expands upon the traditional classification of Aβ (“A”), tau (“T”), and neurodegeneration (“N”), by incorporating a novel inflammatory component (“I”).

**Conclusions:**

The present review outlines the current knowledge on potential neuroinflammatory biomarkers and, importantly, emphasizes the role of longitudinal analyses, which are needed to accurately monitor the dynamics of cerebral inflammation. Such a precise information on time and place will be required before anti-inflammatory therapeutic interventions can be considered for clinical evaluation. We propose that an effective anti-neuroinflammatory therapy should specifically target microglia and astrocytes, while considering the individual ATI(N) status of patients.

## Introduction

Alzheimer’s disease (AD), the predominant type of dementia, accounts for approximately two-thirds of all dementia cases in individuals aged 60 years and older [1]. At present, it affects a staggering 33 million people globally and continues to grow at an alarming rate, with its incidence doubling every 5–10 years [1]. Notably, developing countries play a substantial role in the increasing incidence of new AD cases [2]. This trend may be attributed to the rapid growth of the older population in these regions, which is increasingly affected by dementia. Recently, there has been a significant increase in the focus on disease-modifying therapies that utilize monoclonal antibodies. One notable example is aducanumab, an anti-amyloid agent that received conditional approval from the U.S. Food and Drug Administration (FDA) in 2021 for the treatment of early-stage AD [3]. In 2023, the FDA approved lecanemab, a monoclonal antibody designed to target soluble amyloid-β (Aβ) protofibrils. This groundbreaking approval came after the successful outcomes of a phase III randomized, controlled clinical trial. The results demonstrated, for the first time, that reducing cerebral Aβ plaques through lecanemab led to a noticeable deceleration in cognitive decline over an 18-month treatment period [4]. A recent phase III clinical trial evaluated the efficacy of donanemab, a monoclonal antibody targeting a pyroglutamate form of Aβ, in individuals with prodromal AD and mild dementia due to AD [5]. The study revealed that donanemab effectively slowed both cognitive and functional decline. However, the magnitude of the clinical effects observed with donanemab, along with other similar drugs, such as aducanumab and lecanemab, was limited. This suggests that additional mechanisms, including neuroinflammation [6], tau processing [7], apolipoprotein E (APOE) isoforms imbalance [8], mitochondrial dysfunction [9], and synaptic degeneration [10], should be explored to fully understand and address the pathogenesis of AD. Neuroinflammation refers to the activation of the brain’s innate immune system in response to inflammatory challenges such as injury, infection, toxin exposure, neurodegenerative diseases, or aging. Microglia, the innate immune cells of the central nervous system (CNS), are pivotal in mediating these neuroinflammatory responses [11]. Activated microglia and reactive astrocytes can phagocytize senile plaques or dystrophic neurites, induce intraneuronal inflammatory reactions towards neurofibrillary tangles, and activate the complement cascade in response to vascular amyloid, thereby contributing to cerebral amyloid angiopathy.

Currently, there are 164 ongoing clinical trials in phase I, II, and III, evaluating the effectiveness of 127 distinct drugs. Interestingly, in phase II, approximately 23% of these compounds are specifically targeting inflammatory mechanisms [12]. These efforts highlight the urgent requirement for innovative pharmacological treatments that can effectively prevent or delay the onset of dementia, while also significantly slowing down the progression of the disease. However, inflammatory mechanisms may cycle between inflammation and resolution, and also convert into a chronic type which means that any intervention will require a precise knowledge on the nature and site of inflammation to target.

In this context, the identification of reliable biomarkers of the initial pathological processes assumes paramount significance in disease management during all stages. Conducting biomarker studies becomes imperative in unraveling the intricate interplay between specific immune and/or inflammatory molecules in the development and progression of AD clinical manifestations. Employing longitudinal biomarker studies can unveil varying expression patterns in the initial stages of AD pathology and thus potentially shed light on variances in treatment response [13]. Efforts are currently underway to identify and validate innovative blood-based biomarkers that can effectively reflect the pathophysiological mechanisms associated with AD at a peripheral level. These biomarkers offer several advantages, such as being non-invasive and well-tolerated compared to brain imaging techniques and cerebrospinal fluid (CSF) biomarkers [14], which together will ease longitudinal assessment over years and decades. Plasma levels of hyperphosphorylated tau at position 217 (p-tau_217_) have demonstrated clinical performance that is equivalent to or superior to the FDA-approved CSF tests in detecting brain Aβ pathology [15, 16]. Plasma neurofilament light chain (NfL) protein, which is a scaffolding cytoskeleton protein released when neurons are damaged. Research has shown that plasma NfL concentrations can effectively predict brain imaging biomarkers of neurodegeneration and initial cognitive decline in middle-aged individuals [17].

Currently, there are several fluid biomarkers available for detecting AD dementia development. The AT(N) biomarker framework, which assesses the brain deposition of Aβ (“A”), tau pathology (“T”), and neurodegeneration (“N”), can be further expanded to include neuroinflammatory (“I”) candidate biomarkers, resulting in an ATI(N) system [18, 19]. By monitoring activated microglia and reactive astrocytes, CSF and blood “I” biomarkers enable the tracking of neuroinflammatory processes [20].

In this review, we will provide an overview of the involvement of microglia and astrocytes in the neuroinflammatory processes that impact the AD brain. Furthermore, we will conduct a thorough evaluation of the key studies focusing on biomarkers that track the activation of microglial cells and reactive astrocytes. We will also assess the ability of longitudinal neuroinflammatory biomarker studies to predict the onset of AD and cognitive decline (see Table 1). Lastly, we will analyze the significance of neuroinflammatory biomarkers in the diagnosis of AD and their role in AD clinical trials.

**Table 1:** Main characteristics of selected longitudinal studies on neuroinflammatory biomarkers in AD (n = 27)

References	Study	Mean follow-up period (years)	Population	Biomarkers	Main results
Casati et al., 2018 [82]		2	42 CU 57 MCI 50 AD	Plasma sTREM2 PBMC mRNA TREM2	Higher TREM2 expression at baeseline in *APOE ε4* MCI patients which progressed to AD
Edwin et al., 2020 [77]		3–5	42 CU 231 AD	CSF sTREM2	Higher CSF sTREM2 was associated with slow clinical progression
Morenas-Rodríguez et al., 2022 [78]	DIAN	2.7–3.4	148 ADAD 91 CU	CSF sTREM2	Presymptomatic carriers: increase in CSF sTREM2 correlated with a decrease in Aβ42 Symptomatic carriers: decrease in CSF Aβ42 and Aβ42/40 at baseline independently predicted annual rate of increase in CSF sTREM2
Winfree et al., 2022 [76]	Vanderbilt Memory and Aging Project	4.6	83 CU 72 MCI	CSF sTREM2	High CSF sTREM2 levels predict memory decline
Schmitz et al., 2020 [75]	ADNI	1.5	268 CU	CSF sTREM2 Blood C3	Loss of basal forebrain volume was associated with greater longitudinal accumulation of CSF sTREM2 and higher peripheral blood C3 expression
Craig-Schapiro et al., 2010 [92]		6	198 CU 65 very mild AD 29 mild AD	CSF YKL-40 Plasma YKL-40	CSF YKL-40 was higher in very mild and mild AD-type dementia patients and correlated to higher plasma YKL-40 levels CSF YKL-40/Aβ42 ratio predicted risk of cognitive impairment development
Olsson et al., 2013 [176]	Malmo Cohort	5.7	65 CU 170 NCI 96 AD	CSF YKL-40 CSF sCD14	Baseline YKL-40 and sCD14 increase in MCI patients who converted into vascular dementia
Kester et, 2015 [91]	Amsterdam Dementia Cohort	2.0	37 CU 61 MCI 65 AD	CSF YKL-40 CSF VILIP-1	CSF YKL-40 levels at baseline in MCI and AD patients were higher than CU CSF YKL-40 and VILIP-1 at baseline predicted progression of MCI to AD CSF YKL-40 increased longitudinally in MCI and AD
Lleó et al., 2019 [93]	BIOMARKAPD	2.1	154 CU 75 SCD 128 MCI 110 AD	CSF YKL-40	At baseline, MCI and AD groups showed higher CSF YKl-40 levels than SCD and HC CSF YKL-40 levels increased longitudinally in all groups
Vergallo et al., 2020 [97]	INSIGHT-preAD	1–3	314 SMC	CSF YKL-40 Plasma YKL-40	CSF YKL-40 levels increased longitudinally CSF YKL-40 positively associated with memory performance and negatively with brain Aβ deposition
Pereira et al., 2021 [113]	BioFINDER-2	2	288 CU 141 AD 75 non-AD	Plasma GFAP CSF GFAP	Plasma GFAP associated with both longitudinal Aβ-PET and cognitive decline
Chatterjee et al., 2022 [112]	KARVIAH Cohort	1	206 CU	Plasma GFAP	Increased plasma GFAP levels in Aβ^+^ individuals Plasma GFAP levels showed significant correlations with cognition
Cicognola et al., 2021 [109]		4.7	79 MCI 47 MCI-AD 34 MCI-other	Plasma GFAP	Plasma GFAP predicted conversion to AD Longitudinal plasma GFAP slopes for Aβ^+^ and MCI who progressed to dementia were significantly steeper than those for Aβ^−^ and stable MCI
Silva-Spínola et al., 2023 [111]		5.8	106 MCI	Serum GFAP	At baseline, serum GFAP levels were significantly increased in patients who progressed to AD at follow-up
Verberk et al., 2021 [115]	SCIENCe and Amsterdam Dementia Cohort	3.0	300 CU	Serum GFAP	Higher serum GFAP levels at baseline associated with increased risk of progression to dementia
O’Connor et al., 2023 [110]		3	23 pre-symptomatic ADAD	Plasma GFAP	In pre-symptomatic ADAD participants, plasma GFAP concentration increases over a decade prior to estimated symptom onset
Stocker et al., 2023 [116]	ESTHER Cohort	17	768 CU	Plasma GFAP	GFAP associated with incident AD 9–17 years before diagnosis
Yakoub et al., 2023 [177]	PREVENT-AD cohort	4	373 CU	Plasma GFAP	Female sex showed accelerated increase in plasma GFAP over time compared to males
Bellaver et al., 2023 [114]	TRIAD and MYHAT Cohorts	2.3	1,106 CU	Plasma GFAP	Aβ-dependent tau accumulation occurred only in individuals with plasma GFAP levels above a pre-specified cutoff
Cronjé et al., 2023 [118]	Cardiovascular Health Study	17	1,712 CU	Serum GFAP	Serum GFAP was associated with a hazard ratio of 1.38 for incident dementia, and 2.76 for dementia-specific mortality
Varma et al., 2024 [119]	Baltimore Longitudinal Study of Aging	10	318 CU	Plasma GFAP	In individuals who later converted to AD, elevated plasma levels of GFAP were detected up to a decade before the manifestation of cognitive impairment
Yaffe et al., 2003 [178]	Health, Aging, and Body Composition	2	3,031 CU	Serum IL-6 Serum TNFα Serum CRP	Serum higher IL-6 and CRP levels prospectively associated with cognitive decline
Tan et al., 2007 [179]	Framingham Study	1–2	691 CU	PBMCs IL-1 PBMCs IL-1RA PBMCs TNF-α	Higher IL-1 and TNF-α spontaneous production may predict an AD risk in older individuals
Caldwell et al., 2021 [180]	Center of Biomedical Research Excellence	1	109 CU	Plasma IL-1β Plasma IL-6 Plasma TNFα	In women higher IL-1β at baseline related to poorer verbal learning and delayed recall at 12 months
Arosio et al., 2007 [133]		4	198 CU 48 MCI 193 AD	gDNA TGF-β1	SNPs at codons + 10T/C, + 25 G/C, and the CC genotype associated with an increased conversion from MCI into AD
Fraga et al., 2015 [135]	Pietà Study	1	259 CU	TGF-β1 codon 10T > C	Carriers of at least one T^lower^ allele showed short-term decline in functional performance Individuals with CC^higher^ genotype demonstrated cognitive stability or improvement
Gogishvili et al., 2023 [131]	Amsterdam Dementia Cohort	4	196 CU 210 Demented	CSF TGF-β1	Demented fast-progressor had lower CSF TGF-β1 vs slow-progressor

AD: Alzheimer’s disease; ADAD: Autosomal Dominant AD; ADNI: Alzheimer’s Disease Neuroimaging Initiative; Aβ^−^: Aβ-PET negative; Aβ^+^: Aβ-PET positive; C3: Complement component 3; CRP: C-reactive protein; CU: cognitively unimpaired; DIAN: Dominantly Inherited Alzheimer Network; gDNA: genomic DNA; GFAP: glial fibrillar acidic protein; INSIGHT-preAD: INveStIGation of AlzHeimer’s PredicTors in Subjective Memory Complainers; KARVIAH: Kerr Anglican Retirement Village Initiative in Ageing Health; MCI: mild cognitive impairment; MYHAT: Monongahela-Youghiogheny Healthy Aging Team; PBMCs: peripheral blood mononuclear cells; PET: positron emission tomography; PREVENT-AD: Pre-symptomatic Evaluation of Experimental or Novel Treatments for Alzheimer’s Disease; sCD14: soluble CD14; SCIENCe: Subjective Cognitive Impairment Cohort. SMC: subjective memory complaint; sTREM2: soluble TREM; TRIAD: Translational Biomarkers in Aging and Dementia; VaD: vascular dementia; VILIP-1: visinin-like protein-1; WRAP: Wisconsin Registry for Alzheimer Prevention

## Search strategy and selection criteria

This non-systematic literature review aims to provide an informative overview of the current state of biomarkers for neuroinflammation in AD. The manuscript is based on a selective analysis of high-quality, contemporary articles on neuroinflammation biomarkers in AD. The primary objective is to identify trends and enhance understanding of the current landscape of neuroinflammation biomarkers in AD. References for this review were identified through searches of PubMed databases for peer-reviewed articles published in English between January 1, 2013, and December 31, 2023. The search terms employed included “Neuroinflammation” and “Alzheimer,” “longitudinal studies” and “Alzheimer,” “TREM2” and “Alzheimer,” “GFAP” and “Alzheimer,” and “YKL40” and “neurodegeneration.” Additionally, bibliographies of relevant papers were reviewed. Only papers published in English were considered for inclusion. The final list of references was selected by SL, MG, and AF, and validated by BPI.

## The role of microglia and astrocytes in Alzheimer’s disease pathophysiology

**Fig. 1:**
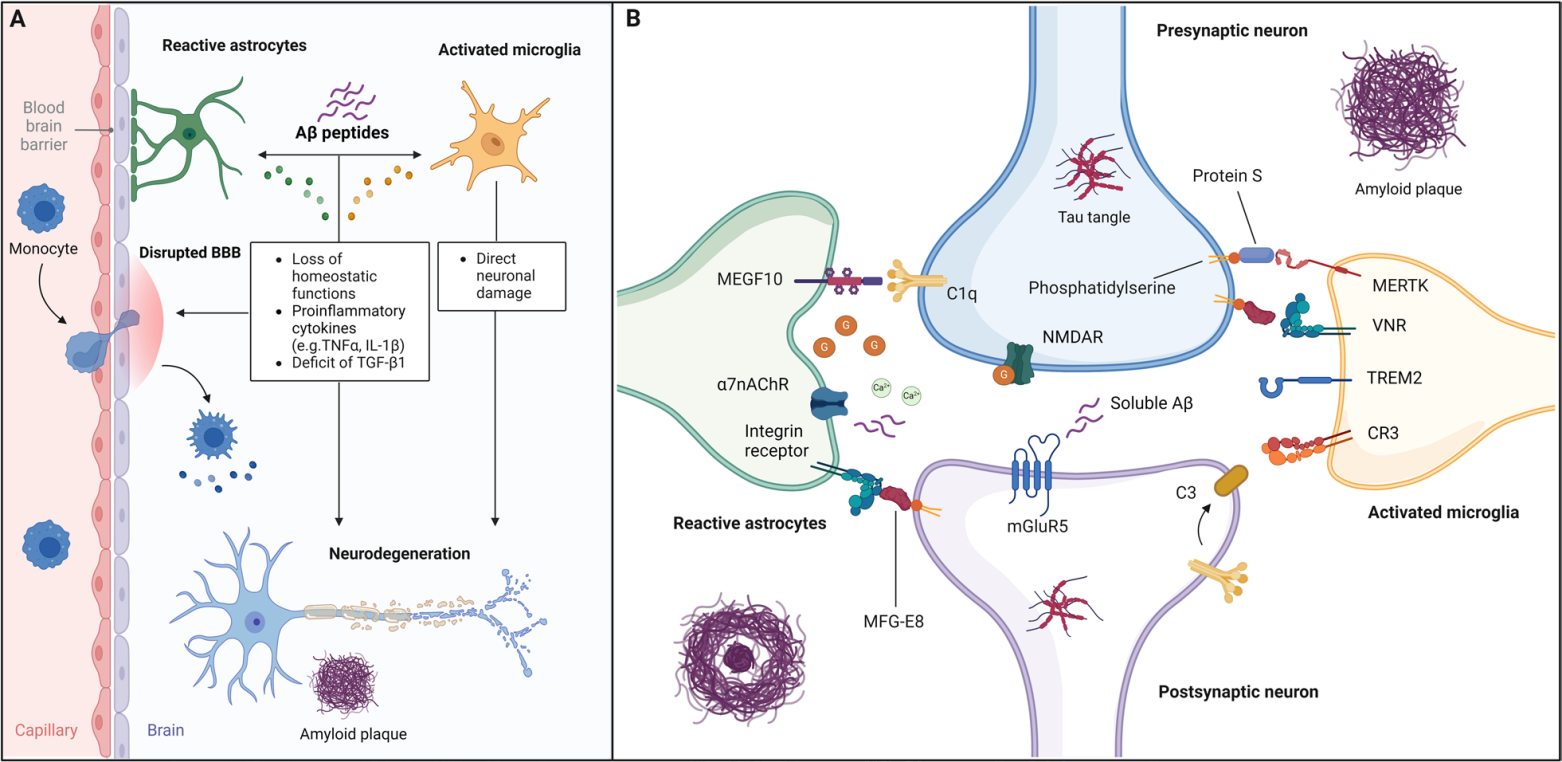
**A** Pathological interactions between glia and neurons in AD. Amyloid-beta species can be recognized by and activate microglia, which contribute to activate astrocytes. These cells release pro-inflammatory cytokines and chemokines and lose their homeostatic functions, leading to disruption of the BBB and neuronal damage. Deficit of anti-inflammatory cytokines (e.g. TGF-β1) also contributes to microglial activation and synaptic dysfunction. Disease associated microglia can also directly eliminate synaptic structures. **B** “Eat-me” signals and the quad-partite synapse in AD. Neuronal eat-me signals are recognized by several phagocytic receptors on both astrocytes and microglia. Some of these signals include milk fat globule-EGF factor 8 protein (MFGE8) and Protein S, both bound to exposed phosphatidylserine, and complement component 3 (C3) and 1q (C1q). Microglia use as cell-surface receptors the complement receptor 3 (CR3), vitronectin receptor (VNR), MER receptor tyrosine kinase (MERTK) and triggering receptor expressed on myeloid cells 2 (TREM2) among others. Astrocytes may also contribute to synaptic engulfment via the binding of C1q to multiple EGF-like domains 10 (MEGF10). Metabotropic glutamate receptor 5 (mGluR5) may also induce synaptotoxicity via calcium dysregulation and complement activation, making the synapses a target for removal by astrocytes and microglia. Interactions between α7 nicotinic acetylcholine receptors (α7nAChRs) and soluble amyloid-β (Aβ) can also contribute to reactive astrogliosis and neuronal death, through the excessive release of glutamate from astrocytes in a Ca2 + -dependent manner. *α7nAChRs* α7 nicotinic acetylcholine receptors, *Aβ* amyloid-β, *AD* Alzheimer disease, *BBB* blood–brain barrier, *C1q* complement component, *C3* complement component 3, *CR3* complement receptor 3 (CR3), *IL* interleukin, *MEGF10* multiple EGF-like domains 10, *MERTK* MER, receptor tyrosine kinase, *MFGE8* milk fat globule-EGF factor 8 protein, *mGluR5* Metabotropic glutamate receptor 5, *TGF-β1* transforming growth factor-beta 1, *TNF* tumor necrosis factor, *TREM2* triggering receptor expressed on myeloid cells 2, *VNR* vitronectin receptor

Astrocytes and microglia, the brain-resident macrophages, are vital components in the development of neural circuits. These dynamic cells establish bidirectional communications with synapses, exerting a profound influence on synaptic function. Contrary to popular belief, synaptic information processing is not solely dependent on neurons. Astrocytes envelop synapses and microglia interact with synapses in an activity-dependent manner [21], collectively contributing to the intricate network of neural connectivity. Prior studies [22, 23] have also demonstrated that astrocytes, microglia, and synapses interact in a “quad-partite” model, where the axon terminal and dendritic spine communicate directly with microglial and astrocytic processes. Disruptions to this quad-partite arrangement can lead to abnormal plasticity, which consequently affects the encoding of information in neuronal circuits (Fig. 1).

Astrocytes play a crucial role in the formation of synapses and regulating the release of neurotransmitters, thereby maintaining the balance of glutamate in the brain. This function of astrocytes is essential for promoting various physiological activities associated with synaptic plasticity and, consequently, cognitive function [24]. Moreover, astrocytes can facilitate neuroinflammatory processes through the release of inflammatory cytokines and chemokines. These cells are also involved in the clearance of Aβ, which subsequently activates them, leading them to encircle senile plaques. This, in turn, contributes to Aβ-induced damage to the BBB, ultimately depriving neurons of their metabolic supply [25].

Microglia cells, which represent approximately 5–20% of all glial cells, serve as the main type of macrophages in the CNS. Their primary function is to regularly survey brain regions for pathogens and cellular debris, ensuring the preservation of neuronal circuits. Additionally, microglia protect and remodel synapses to support brain function [26]. These cells express various receptors that detect both internal and external insults to the CNS. When triggered by pathological factors such as protein aggregates or neuronal death, microglia migrate to the site of injury and initiate innate immune responses [27].

During the inflammatory processes involved in the pathogenesis of AD, there is a transition from the resting to the active functional state of microglia. Inflammation is primarily triggered by the accumulation of Aβ aggregates, including soluble oligomers and insoluble fibrils [28]. In the early stages of AD, Aβ oligomers and fibrils build up in the extracellular space, initiating a pathological cascade that leads to neuronal apoptosis and depletion. Microglia also play a crucial role in clearing Aβ oligomers, fibrils, and dead cells through phagocytosis and by secreting proteolytic enzymes. Additionally, microglia surround plaques and fibrils, forming a barrier that prevents their spread and limits their toxicity [29]. As Aβ deposition becomes increasingly severe, microglia undergo a transition from their normal, homeostatic state to a dysfunctional phenotype. This shift prompts the release of pro-inflammatory factors, oxidative stress, and neuroinflammation, while also reducing the secretion of neurotrophic factors. Ultimately, this leads to synaptic impairment and the exacerbation of neuronal damage [30, 31]. Despite the potential benefits of early activation, chronic activation of microglia by Aβ can be detrimental. This leads to amplified Aβ deposition and prolonged inflammatory processes, ultimately accelerating neurodegeneration. In advanced stages of the disease, microglia can exacerbate AD by circulating proinflammatory cytokines—such as interleukin-1β (IL-1β) and tumor necrosis factor-alpha (TNF-α)—which cause neuronal cell death, as well as by stimulating astrocytes, which can impact neuronal survival [26].

## Biomarkers of neuroinflammation in Alzheimer’s disease

Biomarkers play a crucial role in enhancing our understanding of the molecular mechanisms underlying the onset and progression of AD. Specifically, plasma biomarkers offer a convenient method to evaluate individuals at various stages of the disease continuum, including healthy individuals, those at risk of developing AD, and patients with AD. By monitoring these biomarkers over time, we can gain valuable insights into the individual’s longitudinal progression within the AD spectrum.

As of 2009, genome-wide association studies (GWAS) led to the identification of novel genetic associations, revealing genome-wide statistically significant links between AD and variants within the *CLU*, *PICALM*, and *CR1* genes. Since then, more than 50 risk loci and over 70 gene variants associated with an increased risk of developing sporadic late-onset AD (LOAD) have been identified [32]. These findings underscore the interconnected network of molecular and cellular pathways that significantly influence the progression and pathogenesis of AD. Interestingly, several of these identified variants are intertwined with genes that play a crucial role in immune responses and inflammation. These include, but are not limited to, *TREM2, CD33, PILRA, CR1, MS4A, CLU, ABCA7, EPHA1*, and *HLA-DRB1* [23, 33]. The majority of these genes play significant roles in various functions such as proinflammatory intracellular signaling, cytokines/interleukins/cell turnover, synaptic activity, lipid metabolism, and vesicle trafficking. The crucial involvement of neuroinflammation is supported by extensive GWAS studies that reveal a notable rise in the likelihood of developing LOAD in individuals carrying rare variants of microglial immunoreceptors.

*CD33* encodes a transmembrane receptor expressed on myeloid lineage cells, functioning as a sialic acid-binding immunoglobulin-like lectin that modulates innate immunity. The minor allele of the CD33 SNP rs3865444, which confers protection against AD, has been associated with reduced levels of insoluble Aβ42 in AD brains. In microglial cell cultures, CD33 inhibits the uptake and clearance of Aβ42. Therefore, CD33 inactivation has been shown to mitigate Aβ pathology, suggesting that CD33 inhibition could represent a novel therapeutic strategy for AD [34]. *PILRA* encodes the paired immunoglobulin-like type 2 receptor alpha, a cell surface inhibitory receptor that recognizes specific O-glycosylated proteins. PILRA is expressed on various innate immune cell types, including microglia. It has been proposed that the common *PILRA* missense variant (G78R, rs1859788) protects individuals from AD risk by reducing inhibitory signaling in microglia and microglial infection during herpes simplex virus 1 (HSV-1) recurrence [35]. Complement component (3b/4b) receptor 1 (*CR1*) is a notable candidate gene with a significant connection to AD. Polymorphisms in *CR1* have been reported to be associated with LOAD susceptibility. A recent review identified that the rs6656401 variant in CR1 increases the risk of LOAD [36]. Additionally, a common variant in the membrane-spanning 4-domains subfamily A (*MS4A*) gene, specifically MS4A4A, has been linked to increased CSF sTREM2 concentrations and a reduced risk of AD [37]. The *CLU* gene, which contains several AD-associated intronic SNPs, encodes clusterin, a secretory protein predominantly synthesized in astrocytes. Clusterin levels are elevated in the brain tissues, CSF, and plasma of AD patients and may play anti-amyloidogenic roles [38].

Rare variants of the ATP-binding cassette sub-family A member 7 (*ABCA7*) gene are significantly enriched in patients with AD, while a common *ABCA7* missense variant may confer protection against the disease. Methylation at several CpG sites within the *ABCA7* locus is significantly associated with AD and correlates with amyloid deposition and brain morphology [39]. The *EPHA1* gene encodes ephrin type-A receptor 1 (EphA1), a member of the ephrin receptor subfamily within the protein-tyrosine kinase family. Ephrin receptors and related proteins have been implicated in mediating immune cell recruitment. The P460L variant of the *EPHA1* gene is associated with an increased risk of LOAD and has recently been shown to impact immune responses and blood vessel function in the brain [40]. HLA-DRB1 is part of the human leukocyte antigen (HLA) class II beta chain paralogues. This class II molecule is a heterodimer composed of an alpha (DRA) and a beta (DRB) chain, both of which are membrane-anchored. HLA-DRB1 plays a pivotal role in the immune system by presenting peptides derived from extracellular proteins. Specific HLA-DRB1*04 alleles have been shown to confer protection against AD [41]. Below, we provide an in-depth description of the TREM2 protein and other significant neuroinflammatory proteins.

### Soluble triggering receptor expressed on myeloid cells 2 (sTREM2)

TREM2, a cell surface receptor, is predominantly expressed on microglial cells. Its primary function includes enhancing the phagocytic capabilities of microglia and macrophages while also modulating inflammatory signaling. TREM2 transduces its intracellular signaling through the adapter protein DAP12 [42]. The binding of TREM2 to ligands, including anionic lipids, lipoproteins, and Aβ, initiates downstream signaling cascades that promote microglial survival, proliferation, chemotaxis, and phagocytosis [43, 44]. The significance of TREM2 in the pathogenesis of AD was underscored by the discovery of a specific heterozygous mutation, R47H, which substantially elevates the risk of developing LOAD [45, 46]. Subsequent research has established that TREM2 activation in microglia plays a crucial role in the pathological processes underlying AD [47]. This receptor also plays a pivotal role in regulating microglial-related activities, particularly in response to the presence of Aβ plaques and tau tangles [48]. A soluble form of TREM2 (sTREM2), which is generated through the proteolytic cleavage of the receptor found on the surface of cells, has the ability to promote microglial activation. Proper receptor ligation is anti-inflammatory and facilitates microglial survival.

*TREM2* gene variants have been found to increase the risk of developing AD by impairing the ability of microglia cells to effectively clear Aβ and disrupting the normal proinflammatory response of these immune cells [49]. Currently, approximately 50 variants of the *TREM2* gene have been studied in relation to AD [50]. The rare *R47H* variant of *TREM2* (rs75932628) has been linked to a two- to three-fold increase in the risk of developing AD in European and North American (Caucasian) populations [51–53]. The *R47H* variant is believed to contribute to a loss of TREM2 function, leading to a reduction of microglia and an increase in neuritic dystrophy at the site of Aβ deposition [54]. A meta-analysis involving more than 73,000 participants found that individuals carrying this variant had a four-fold higher risk of developing AD compared to non-carriers, which is similar to the effect size of the *APOE ε4* variant [55]. Additionally, it has been suggested that the *R47H* variant may be associated with an earlier age at AD onset [56]. However, this variant is extremely rare or undetectable in African-American [57] and Asian populations [58, 59]. Another variant, *R62H* (rs143332484) [60], has been associated with an increased risk of AD in individuals of European descent [61]. Interestingly, patients with AD harboring *TREM2* risk variants show an abundance of autophagic vesicles in their microglia [62].

An additional variant, *H157Y* (rs2234255), is located at the TREM2 site and is cleaved by two α-secretases (a disintegrin and metalloproteinase 10 and 17 [ADAM-10 and ADAM-17]), resulting in increased shedding of TREM2 and reduced cell surface expression of the receptor [63]. Unlike the *R47H* and *R62H* variants, *H157Y* is more frequently observed in Asians and is particularly associated with an increased risk of AD in a Han Chinese cohort [64, 65]. Prior studies conducted on Caucasian, Japanese, and African-American cohorts did not find any significant association between this variant and AD. However, a comprehensive meta-analysis revealed a strong correlation with an odds ratio of 3.65 [65]. Additionally, further analyses conducted on Chinese cohorts identified the presence of the p.Ala130Val and p.Ala192Thr variants, specifically observed in cases of LOAD. Another variant, p.Ser183Cys, was found to be more prevalent among Chinese patients with AD [50]. The presence of multiple variants associated with a higher susceptibility to developing AD in Chinese and African-American populations highlights the presence of diverse mutations across different cohorts and ethnic groups. This underscores the need to investigate various ethnic populations to discover specific disease risk variants and explore potential associations between these variants and specific disease phenotypes [50]. Notably, individuals carrying *TREM2* variants exhibit an earlier onset of the disease and experience faster cerebral atrophy. Therefore, identifying *TREM2* carriers can be valuable in improving patient stratification for clinical trials and supporting the development of personalized therapeutic approaches [33].

In general, the published literature has shown a slight increase in CSF sTREM2 concentrations in patients with AD [66–68] and individuals with mild cognitive impairment (MCI) [69] compared to CU controls. However, other studies did not find significant differences in CSF sTREM2 levels between AD or MCI participants and CU controls [70]. Notably, increasing CSF sTREM2 concentrations were associated with changes in brain structure, specifically an increase in grey matter volume in regions such as the bilateral inferior and middle temporal cortices, precuneus, supramarginal and angular gyri, in individuals with MCI. This suggests that sTREM2 may play a role in modulating neuroinflammatory responses to early neurodegeneration [71].

Despite the fact that CSF sTREM2 values are higher in patients with AD compared to CU controls, this potential biomarker lacks sufficient discriminatory power. Specifically, it falls short of the 80–90% range required in clinical diagnostic practice to effectively differentiate between patient groups [69]. However, the increase in CSF sTREM2 concentrations during the early stages of AD may indicate a change in microglial activation status due to neurodegeneration. This suggests that sTREM2 exhibits a dynamic response linked to microglial activity as the disease progresses. These findings highlight the potential of CSF sTREM2 as a biomarker for tracking the progression from preclinical AD/MCI to AD dementia [68, 72]. Consequently, it may also serve as a valuable biomarker in clinical trials focusing on secondary AD prevention. The dynamics of CSF sTREM2 should be investigated in relation to the key pathophysiological mechanisms of AD, namely Aβ and tau pathologies. Decreased levels of CSF sTREM2 are linked to Aβ pathology [73] while increased levels are associated with tau-related neurodegeneration [74]. In a cross-sectional study of 127 individuals with autosomal dominant AD (ADAD) mutations, it was observed that CSF sTREM2 concentrations began to rise five years before symptom onset, but quite a long time after the accumulation of Aβ in the brain [72].

Significant insights have also been gained from longitudinal studies on CSF sTREM2. For instance, a 1.5-year study involving 268 CU individuals with initial brain Aβ deposition revealed that a decline in basal forebrain volume was associated with a greater accumulation of CSF sTREM2 over time [75]. In another longitudinal study spanning 4 years and including 72 individuals with MCI, it was found that CSF sTREM2 levels correlated with the progression of CSF Aβ and tau [76]. Additionally, a 3-year study on 231 AD patients showed that higher CSF sTREM2 levels at baseline were linked to slower clinical progression [77]. Finally, a 3-year study on 148 pre-symptomatic ADAD patients indicated that higher annual rates of increase in CSF sTREM2 corresponded to a reduced rate of cognitive decline [78]. These studies suggest that sTREM2 should be considered not an early marker, but indicative of the MCI to AD conversion, although further studies are needed to validate its role as biomarker in this scenario.

Limited reports have investigated the dynamics of TREM2 in blood. However, the available research suggests that both TREM2 mRNA and protein expression levels are elevated in monocytes of patients with AD compared to controls. Furthermore, these increased levels were found to be inversely correlated with cognitive performance, as measured by the Mini-Mental State Examination (MMSE). A positive correlation between TREM2 mRNA and protein expression was observed in monocytes. In addition, a tendency towards upregulation of TREM2 protein was also noticed in granulocytes and plasma [79]. Other studies have demonstrated higher expression levels of TREM2 mRNA in leukocytes of patients with AD than in controls, which were found to be associated with cognitive decline and hippocampal atrophy [80, 81]. In a longitudinal study involving 57 individuals with MCI, higher plasma TREM2 levels and mRNA expression were detected in peripheral blood mononuclear cells (PBMCs) of *APOE ε4* positive individuals who later developed AD during the two-year follow-up period [82]. Another investigation focusing on peripheral leukocytes revealed higher expression levels of TREM2 in LOAD compared to early-onset AD. Interestingly, the expression of this receptor was markedly increased in LOAD individuals who carried the *APOE ε4* allele [83].

### Chitinase-3-like protein 1 (YKL-40)

The glycoprotein YKL-40, also known as chitinase-3-like protein 1 (CHI3L1), is detected primarily in various cell types, including macrophages, chondrocytes, fibroblasts, vascular smooth muscle cells, endothelial cells, and even certain cancer cells [84]. Expression is most prominent in reactive astrocytes [85]. While the exact physiological role of YKL-40 is still debated, there is consensus regarding its involvement in tissue remodeling and renovation during inflammation, as well as in angiogenic mechanisms that affect the infiltration, differentiation, and maturation of macrophages. Consequently, YKL-40 is considered a candidate biomarker for inflammation and endothelial dysfunction [84], a specific biomarker for human macrophage activation/differentiation and its expression is induced in reactive astrocytes by the presence of activated macrophages [86]. A critical review of the literature has identified YKL-40 as a pathophysiological biomarker indicating the activation of glial cells, including astrocytes and microglia, which are associated with tau pathology [87, 88]. In addition, prior studies have shown that CSF levels of YKL-40 are linked to biomarkers of neurodegeneration (total tau, t-tau), damage to large-caliber myelinated axons (NfL), tau-mediated toxicity (p-tau), and synaptic damage (neurogranin, SNAP-25) in various neurodegenerative diseases [89].

Interest in YKL-40 in AD was sparked by initial observations that CSF levels of YKL-40 were significantly higher in individuals with AD and MCI compared to controls [90, 91]. Notably, early histopathological studies have demonstrated that YKL-40 expression is localized near Aβ plaques and tau neurofibrillary tangles [92]. YKL-40 can effectively differentiate patients with overt dementia from CU individuals [91–94]. Secondly, YKL-40 serves as a predictor of cognitive decline, allowing for the identification of individuals progressing from preclinical AD to prodromal AD and later stages of dementia. Finally, it aids in distinguishing individuals with MCI who will convert to AD from those who will remain stable at five years [86]. However, it should be noted that CSF YKL-40 is not clinically useful in differentiating the characteristic AD phenotype (i.e., amnestic syndrome of hippocampal type) from other atypical presentations of AD [95]. In addition, patients exhibited a significant correlation with the corresponding CSF values. Significantly, patients in a 6-year longitudinal study by Craig-Schapiro and colleagues (2010) exhibited a strong association between YKL 40 levels in CSF and plasma [92]. Moreover, plasma YKL-40 concentrations were found to be elevated in individuals with very mild and mild AD dementia (CDR = 0.5 and CDR = 1, respectively) compared to control individuals (CDR = 0) [92]. Another study discovered a significant increase in plasma YKL-40 levels in patients with early AD than in individuals with MCI and healthy older controls. Moreover, YKL-40 demonstrated a positive correlation with neuropsychological test results in both MCI and early AD [96]. In a longitudinal study conducted by Vergallo et al. (2020) [97], it was found that plasma YKL-40 concentration can serve as a valuable biomarker to assess the severity of AD. The investigation focused on a cohort of CU individuals at risk for AD and revealed a positive association between plasma YKL-40 and episodic memory performance (assessed using the Free and Cued Selective Rating Test). Conversely, a negative association was observed between plasma YKL-40 and brain Aβ accumulation [97]. These findings suggest that glia activation, reflected by elevated YKL-40 levels, may have a potentially protective effect on initial brain Aβ deposition and neuronal homeostasis, without causing clinical harm. Moreover, the study also observed a sexual dimorphism, with men exhibiting higher YKL-40 concentrations than women [97]. Another study investigating biomarker differences across different ethnicities reported elevated plasma YKL-40 concentrations specifically in Hispanic women with prodromal AD compared to both CU controls and prodromal AD participants of African or Caucasian origins. This emphasizes the impact of potential modulating factors on the variability of YKL-40 levels [98].

YKL-40 has also emerged as a compelling candidate biomarker for investigating the clinical evolution of AD. Its potential role in clinical trials lies in its ability to track the dynamics of glial neuroinflammatory mechanisms in relation to neurodegeneration. According to Baldacci et al. (2017), CSF YKL-40 exhibits a correlation with elevated levels of CSF t-tau, even in asymptomatic and preclinical AD individuals. This suggests an early association between YKL-40 and tau protein during the course of neurodegeneration [86].

In conclusion, exploring the connection between YKL-40 and AD features could shed light on the intricate relationship between Aβ dysmetabolism, neuronal activity, and neuroinflammation. YKL-40 thus represents a promising biomarker for accurate classification of neuroinflammatory phenotypes, facilitating advancements in neuroinflammatory clinical trials [97].

### Glial fibrillary acidic protein (GFAP)

Astrogliosis refers to the abnormal activation and proliferation of astrocytes that occurs in response to initial brain damage [99]. This process leads to significant cellular, molecular, and functional changes. In acute brain injuries—as well as in AD, other neurodegenerative diseases, and low-grade astrocytoma—astrocytes adopt a reactive phenotype [99]. Transcriptomic analyses of human patients and disease models have demonstrated the presence of multiple putative reactive astrocyte sub-states. In CNS disorders, there is a marked upregulation and reorganization of intermediate filament proteins, leading to the formation of an intricate network comprising various isoforms of GFAP (ten isoforms), vimentin, synemin, and nestin [100]. GFAP, a type III intermediate filament protein, serves as a key cytoskeletal component in astrocytes. In AD, astrocytes exhibit a complex response to both neurofibrillary tangles and Aβ plaques, which may exert either neuroprotective or deleterious effects [101]. The capacity of astrocytes to colocalize with Aβ plaques in the AD brain has been demonstrated using labeled tracers [102]. Moreover, studies have reported a significant correlation between GFAP expression levels and the density of Aβ plaques in the hippocampus and entorhinal cortex of AD brains [103]. Interestingly, elevated concentrations of GFAP within the CSF have been reported in patients with AD and other forms of dementia compared to healthy individuals [104]. An increase in plasma GFAP concentrations was also noted in both early-onset AD and LOAD [105, 106]. This increase was positively correlated with the extent of white matter injury, as determined through the quantification of white matter hyperintensities [105, 106], whereas cognitive function assessed with MMSE showed an inverse association [106]. When combined with plasma Aβ_1-42_/Aβ_1-40_ ratio, the *APOE ε4* status, and/or age, the diagnostic value of plasma GFAP was found to be increased [107–109].

The potential of blood GFAP as a biomarker for AD has been significantly highlighted by longitudinal investigations. A study conducted over 3 years on 23 asymptomatic AD patients revealed an increase in plasma GFAP concentrations in mutation carriers compared to non-carrier controls. This suggests that plasma GFAP alterations can be detected up to a decade before the onset of AD clinical symptoms [110]. Additionally, a 6-year longitudinal study on 106 individuals with MCI found that baseline serum GFAP levels were significantly higher in patients who progressed to AD at follow-up [111]. In a cross-sectional analysis conducted in preclinical AD, a plasma biomarker panel consisting of GFAP, p-tau_181_, and p-tau_231_ exhibited increased levels in CU Aβ-positron emission tomography (PET)**-**positive individuals compared to those who tested negative [112]. The study subsequently confirmed the longitudinal predictive value of two of the three biomarkers (GFAP and p-tau_181_), emphasizing their diagnostic and monitoring potential in preclinical AD [112]. In a 2-year, longitudinal study, researchers examined 288 CU individuals and 141 patients. The study found that plasma GFAP levels were associated with both longitudinal Aβ-PET deposition and cognitive decline [113]. Another extensive 2-year longitudinal study involving 1,106 CU participants revealed that Aβ-dependent tau accumulation occurred only in individuals who tested positive for astrocyte reactivity—which was defined by plasma GFAP levels above a specified cutoff (mean + 2.0 standard deviations of controls without Aβ pathology) [114]. These data suggest that secondary astrocytosis caused by Aβ aggregation might promoting tau accumulation, although further longitudinal studies are needed. A 5-year longitudinal study involving 169 individuals with MCI showed that higher baseline plasma GFAP concentrations were associated with the progression to AD and faster rates of cognitive decline [109]. Another 3-year longitudinal study conducted on 300 CU older individuals found that higher serum GFAP levels at baseline were linked to an increased risk of incident dementia [115]. An analysis conducted within the ESTHER cohort, a German population-based study of older individuals living in the community, revealed a substantial early association (spanning between 9 and 17 years prior to clinical diagnosis) of plasma GFAP with the incidence of AD. Notably, this association was found to be significantly earlier than that of NfL and p-tau_181_, which were typically associated within approximately 9 years of diagnosis [116]. These results suggested that GFAP may serve as a more effective prognostic biomarker for incident AD dementia compared to NfL [115]. The diverse prognostic values of these cytoskeletal proteins are believed to stem from different underlying mechanisms. Notably, NfL is released into the bloodstream following axonal degeneration, while increased levels of GFAP are a response to damage triggered by Aβ and tau aggregates [101]. Continual activation of astrocytes in response to this damage leads to a pro-inflammatory neurotoxic state, which further exacerbates neurodegeneration [117].

A recent 17-year longitudinal study involving 1712 CU participants found that serum GFAP levels at baseline were associated with a hazard ratio of 1.38 (95% confidence interval = 1.15–1.66) for incident dementia and 2.76 (95% confidence interval = 1.73–4.40) for dementia-specific mortality, supporting the notion that circulating GFAP can be a valuable tool for assessing dementia risk and prognosis [118]. In another recent study involving 318 CU participants, including 158 individuals who later converted to AD and 160 who remained cognitively unimpaired, the authors observed elevated plasma levels of GFAP in AD-converters up to 10 years before the onset of cognitive impairment [119]. This finding suggests that increased astrocyte reactivity, as indicated by higher GFAP levels, is an early event in the progression of blood biomarker changes during the preclinical stage of AD. Taken as a whole, these results indicate that GFAP holds promise an early blood-based biomarker for reactive astrogliosis associated with Aβ pathology in preclinical AD. Consequently, this marker could be used to identify individuals at risk of AD before the onset of clinical symptoms [108].

### Transforming growth factor-beta 1 (TGF-β1)

Recent studies indicate that a deficiency in anti-inflammatory cytokines, particularly transforming growth factor-β1 (TGF-β1), in the brains of patients with AD significantly contributes to microglia activation and neuroinflammation, thereby playing a crucial role in the pathophysiological mechanisms underlying cognitive decline in AD. [120, 121].

Building on this evidence, researchers have explored the potential of TGF-β1 as a novel biomarker for AD [120]. The deficit of TGF-β1 can contribute to neurodegeneration through multiple mechanisms. Notably, TGF-β1 plays a constitutive role in suppressing inflammation and regulates the degree of microglial activation in the CNS in an age-dependent manner [122]. TGF-β1 also plays a pivotal role in synaptic plasticity and memory formation by facilitating the transition from early to late hippocampal long-term potentiation [123] and stimulating the uptake of Aβ by microglia [122]. Notably, numerous studies have shown that the TGF-β1 signaling pathway is selectively impaired in the early stages of AD, leading to microglia activation, neuroinflammation, increased neuronal vulnerability to Aβ oligomers, hippocampal atrophy, and cognitive decline [121, 124]. In addition, the AD brain exhibits a reduced expression of TGF-βR2, a specific receptor which correlates with the pathological hallmarks of the disease [125]. When evaluating the potential of TGF-β1 as a novel biomarker for early AD, it is essential to consider the differing results obtained from its measurement in the plasma *versus* the CSF of AD patients. AD patients display decreased concentrations of active and inactive forms of TGF-β1 in their plasma [126] as well as a decline in its secretion from PBMCs [127]. TGF-β1 levels were found to be elevated in the CSF and brain of AD patients compared to non-demented individuals, and positively correlated with the extent of cerebrovascular Aβ deposition [128].

Consequently, patients with AD exhibit elevated levels of TGF-β1 in their CSF [129], while decreased concentrations of both total and cleaved (active) forms of this molecule have been observed in their plasma [130]. These seemingly contradictory findings can be clarified by longitudinally assessing TGF-β1 levels at various stages of AD in future prospective long-term observational studies. We hypothesize that elevated levels of TGF-β1 may act as a neuroprotective factor in the early phases of AD pathogenesis, while decreased levels contribute to neurodegeneration and cognitive decline in individuals with MCI [121]. Interestingly, a study on patients with dementia found lower CSF concentrations of TGF-β1 in individuals with fast disease progression compared to those with slower progression [131]. Genetic investigations have provided only preliminary and partial evidence regarding the deficit of TGF-β1 in AD [121].

The TGF-β1 gene is located on chromosome 19q13.1–3 and contains multiple functional single nucleotide polymorphisms (SNPs) in the upstream and transcript regions [132]. Two studies demonstrated that the SNPs at codons + 10 (T/C) and + 25 (G/C), as well as the CC genotype of the *TGF-β1* gene, which are associated with reduced TGF-β1 levels, have been linked to an increased conversion from MCI to AD [133, 134], whereas another research involving oldest-old individuals aged over 75 demonstrated that carriers of at least one minor T allele displayed a significant decline in cognitive and functional performance in the short-term, while those harboring the CC genotype of the TGF-β1 codon + 10 T > C polymorphism remained stable [135]. Building on the evidence obtained from AD patients, various studies have been conducted in animal models of AD to validate the role of TGF-β1 as both a novel biomarker and a potential pharmacological target [136]. It has been hypothesized that the selective deficit of the canonical TGF-β1/Smad pathway in AD may impair the cross-talk between astrocytes and microglia, subsequently leading to microglia-mediated neurodegeneration [136]. *APOE ε4* impairs the microglial response in AD by inducing TGFβ1-mediated checkpoints, suggesting a neurobiological link between *APOE ε4* and the deficit of TGFβ1 signaling in the disease process [137]. Despite conflicting findings regarding TGF-β1 levels at various stages of AD, this anti-inflammatory cytokine has emerged as one of the leading 20 CSF candidate biomarkers associated with the rate of cognitive decline in dementia patients, as demonstrated in longitudinal studies [131]. To validate the role of TGF-β1 as a novel biomarker in early AD, future prospective long-term observational studies are essential.

### Other neuroinflammatory biomarkers

Numerous cytokines and chemokines, commonly linked to inflammation, vascular injury, and angiogenesis, have emerged as potential neuroinflammatory biomarkers. Among these, eight can be measured in serum—including basic fibroblast growth factor (bFGF), C-reactive protein (CRP), interleukin-16 (IL-16), soluble fms-like tyrosine kinase-1 (sFLT-1), soluble intercellular adhesion molecule-1 (sICAM1), the Tie-2 receptor tyrosine kinase, vascular endothelial growth factor-C (VEGF-C), and vascular endothelial growth factor-D (VEGF-D). Three others, interleukin-15 (IL-15), monocyte chemoattractant protein-1 (MCP-1), and sFLT-1, are quantifiable in the CSF[138]. The addition of these neuroinflammatory biomarkers to traditional AD biomarkers improved diagnostic accuracy by 13.9% and 12.5%, respectively, in older individuals with cognitive decline [139]. However, further studies are needed to confirm their utility in clinical settings.

A multicenter study has highlighted the significance of complement dysregulation as a potential predictor of disease progression in MCI [140]. Specifically, the results revealed higher levels of Factor B enzyme and lower levels of Factor H regulator in MCI progressors as compared to non-progressors. Collectively, these findings suggest that the dysregulation of the complement system’s amplification loop may act as an early event that predisposes to AD progression [140].

Multiple meta-analyses have explored the potential of various molecules as biomarkers for AD. In one meta-analysis, which included 54 studies measuring cytokine concentrations (40 in peripheral blood and 14 in the CSF), patients with AD exhibited higher concentrations of IL-6, TNF-α, IL-1β, TGF-β, IL-12, and IL-18 in peripheral blood, and elevated levels of TGF-β in CSF, compared to healthy controls [141]. Another meta-analysis comprising 175 studies on peripheral blood revealed increased levels of IL-1β, IL-2, IL-6, IL-18, interferon-γ, homocysteine, high-sensitivity CRP (hs-CRP), C-X-C motif chemokine-10, epidermal growth factor, vascular cell adhesion molecule-1, TNF-α converting enzyme, soluble TNF receptors 1 and 2, α1-antichymotrypsin, as well as decreased concentrations of IL-1 receptor antagonist and leptin in patients with AD compared to controls [142].

A comprehensive meta-analysis of 170 studies revealed significantly elevated blood concentrations of various proteins including hs-CRP, IL-6, soluble tumor necrosis factor receptors 1 and 2 (sTNFR1 and TNFR2), α1-antichymotrypsin (α1-ACT), IL-1β, and soluble CD40 ligand (sCD40L) in patients with AD compared to controls. Additionally, the CSF concentrations of certain proteins such as IL-10, MCP-1, TGF-β1, sTREM2, YKL-40, α1-ACT, nerve growth factor, and visinin-like protein-1 (VILIP-1) were also found to be higher in AD [143]. Furthermore, patients with MCI exhibited increased peripheral blood concentrations of sTNFR2, IL-6, MCP-1, and decreased concentrations of IL-8. The authors also observed elevated CSF concentrations of YKL-40, VILIP-1, and sTREM2 in MCI patients compared to controls. Finally, patients with AD were found to have increased peripheral blood concentrations of sTNFR1 and sTNFR2 compared to those with MCI [143]. Another meta-analysis comprising 88 studies found increased levels of CRP, IL-1β, IL-2, IL-6, IL-12, IL-18, MCP-1, MCP 3, IL-8, and interferon-γ-inducible protein 10 (IP-10) in patients with AD [144]. These findings were at least in part consistent with a separate meta-analysis involving 13 studies that indicated an association between inflammatory candidate proteins—including CRP, IL-6, α1-ACT, lipoprotein-associated phospholipase A2, and fibrinogen—and an increased risk of all-cause dementia, although these biomarkers were not specific to AD [145].

## The role of neuroinflammatory biomarkers in Alzheimer’s disease diagnosis and therapy

### Role of neuroinflammatory biomarkers in the diagnostic work-up of Alzheimer’s disease

The definitive diagnosis of AD—in the absence of neuropathological confirmation—continues to pose a significant challenge. Despite extensive research into the molecular and biological mechanisms underlying the disease, the effective identification of AD remains an elusive task. This poses limitations on therapeutic interventions, as they are often initiated after the onset of symptoms. In this scenario, there is a growing interest in studying and treating the prodromal stages of AD. One unresolved question in understanding AD pathophysiology is why a considerable percentage of brain Aβ-positive CU individuals do not develop detectable downstream tau pathology and subsequent clinical decline. Recent research has shown that elevated levels of phosphorylated tau in the blood are associated with Aβ accumulation in the brain only in individuals with abnormally high blood levels of GFAP [114]. These findings suggest that astrocyte reactivity, evaluated through the measurement of plasma GFAP, is a significant precursor event that connects brain Aβ accumulation to the onset of tau pathology. This connection may have important implications for the biological characterization of preclinical AD. Furthermore, considering that neurotoxic reactive astrocytes are stimulated by activated microglia [117], the availability of a biomarker for activated microglia becomes essential in identifying individuals who are at a higher risk of developing AD. While CSF sTREM2 and 18 kDa translocator protein (TPSO)-PET imaging are effective for identifying activated microglia, they do not inform about the precise molecular and functional cellular status. Furthermore, a reliable blood biomarker remains elusive. The availability of blood-based biomarkers for both activated microglia and reactive astrocytes could assist in the clinical recognition of MCI and AD, or even earlier stages[138, 146]. Moreover, these biomarkers could facilitate tracking disease progression over time in patients as a part of therapeutic strategies and potentially provide personalized drug targets for early intervention in MCI and AD cases.

### Role of neuroinflammatory biomarkers in anti-Alzheimer’s disease trials and individualized therapy

**Fig. 2:**
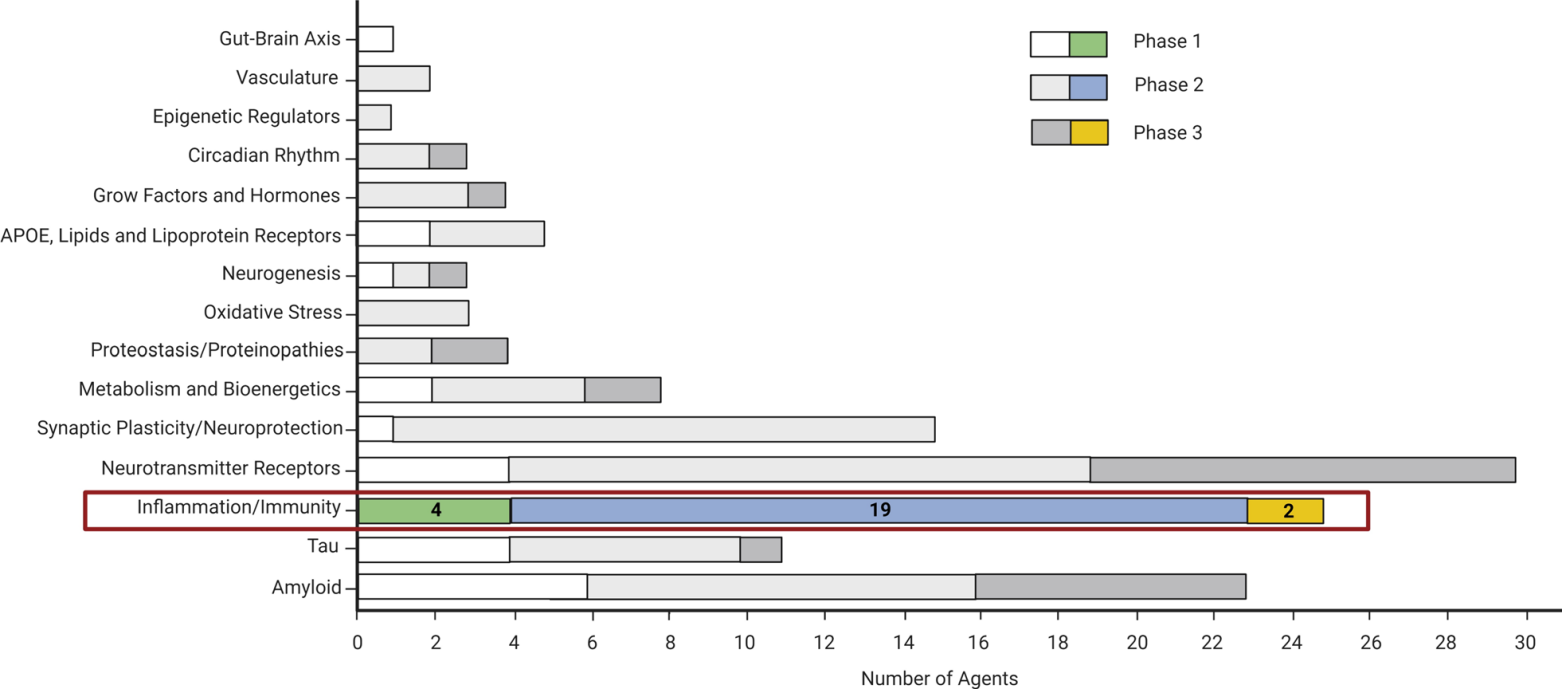
Presently ongoing clinical trials in AD by mechanism of action of the tested agents. Twenty-five trials are testing anti-neuroinflammatory agents. *AD* Alzheimer disease, *APOE* Apolipoprotein E. Modified from Cummings et al. Alzheimers Dement (N Y). 2024 [12]

Targeting neuroinflammation may prove to be an extremely effective strategy for AD prevention and therapy during the preclinical stage before significant neuronal loss occurs. Figure 2 shows the number of ongoing AD clinical trials with anti-neuroinflammatory agents [12].

Overall, there are 25 ongoing clinical trials targeting neuroinflammation in AD: four in phase 1, 19 in phase 2, and two in phase 3 [masitinib (tyrosine-kinase inhibitor) and NE3107 (insulin-sensitizing agent)]. Promising results have been observed in several phase I/II clinical trials that targeted TNF-α, TREM2, or CD33. We strongly advocate for the utilization of neuroinflammatory biomarkers, such as blood GFAP for tracking reactive astrocytes and CSF-sTREM2 for monitoring microglial activation, throughout clinical trials [147]. In our view, these biomarkers present a significant potential for tracking disease progression within the AD continuum in the field of drug development, including trials with compounds not directly impacting biological inflammatory targets. Accordingly, blood GFAP concentrations have already been successfully employed as a biomarker in clinical trials evaluating anti-Aβ monoclonal antibodies, such as lecanemab [148] and donanemab [149]. In general, we are confident that adopting a biomarker-guided strategy for AD treatment, which tailors specific interventions to relevant molecular pathways, will enhance therapeutic effectiveness, as witnessed in the field of oncology. This approach has already seen application in the NSAID treatment of AD. A *post-hoc* analysis of a trial of naproxen and rofecoxib for mild-to-moderate AD demonstrated that those who responded favorably exhibited a distinct plasma neuroinflammatory profile (TNF-α, CRP, IL-6 and IL-10) [150]. These results imply that the use of anti-inflammatory drugs for AD should be reserved for patients who show clear signs of systemic inflammation. A Phase II trial, conducted more recently, with donanemab—a potent anti-Aβ(p3-42) monoclonal antibody—exclusively enrolled patients who, as confirmed by [^18^F]flortaucipir-PET scans, exhibited pathologic tau deposition (> 1.10 SUVR), but with quantitative tau levels below a specific upper threshold (1.46 SUVR) [151]. This strategic approach was undertaken to address concerns surrounding the limited efficacy of donanemab in advanced disease situations, as suggested by the presence of extensive tau pathology. Another significant investigation is the AHEAD prevention study, currently in its fourth year. This research is analyzing the effects of lecanemab in CN participants at risk of developing AD due to the presence of brain Aβ accumulation, as evidenced by Aβ-PET scans [152]. This investigation is divided into two sub-studies (AHEAD 45 and AHEAD 3). AHEAD 45 focuses on participants exhibiting elevated brain Aβ-PET pathology, specifically above 40 Centiloids, measured during the screening phase. In contrast, AHEAD 3 is being conducted on individuals with intermediate brain Aβ pathology levels, defined as 20 to 40 Centiloids, also measured during the screening process. The primary purpose of this comprehensive study is twofold. Firstly, it aims to determine if lecanemab treatment outperforms a placebo in modifying baseline Preclinical Alzheimer Cognitive Composite 5 (PACC5) after 216 weeks of treatment (A45 Trial). Secondly, it seeks to establish if lecanemab treatment is superior to a placebo in mitigating brain Aβ accumulation, as measured by PET scans, following 216 weeks of treatment (A3 Trial) [152]. We posit that the integration of a reliable blood biomarker of neuroinflammation is essential for the effective and predictive ATN(I) categorization of the AD continuum. During the pathological progression of AD, a pivotal moment occurs when innate immune and glial cells begin to sustain an excessively expressed chronic inflammatory response. This process acts in synergy with the accumulation of Aβ and tau proteins, driving synaptotoxicity and neurodegeneration in a self-perpetuating cycle. The precise timing of this neuroinflammatory shift in individual cases remains elusive, possibly explaining why past clinical trials exploring anti-inflammatory compounds have failed to yield successful results. Plasma GFAP displays a compelling ability to predict individual clinical AD risk and is thus suggested as a potential preliminary screening tool for AD risk stratification in the older adult population [153]. The presence of plasma GFAP “positivity” may be a straightforward indicator for initiating a comprehensive therapy. This treatment—which would combine anti-inflammatory drugs with agents that modulate either Aβ or tau—may be particularly applicable to those with preclinical AD or individuals at risk of developing AD.

Risk stratification tools tailored to each individual are crucial for applying precision medicine principles in AD. Blood biomarkers for AD offer a promising strategy that is both time and cost-effective. They hold potential to identify and categorize patients at risk of developing AD, thereby enhancing the screening procedures for potential participants in AD clinical trials. Additionally, these biomarkers can significantly improve patient management in clinical settings. This includes making informed decisions about treatment, such as choosing a disease-modifying therapy based on altered biomarker profiles, or referring patients to specialized memory clinics for focused care [19].

Tracking neuroinflammatory biomarkers could also be crucial for monitoring amyloid-related imaging abnormalities (ARIA), a significant adverse event associated with anti-Aβ monoclonal antibodies, including lecanemab and donanemab [154]. These ARIA manifest as brain edema (ARIA-E), microbleeds, and occasionally large brain hemorrhages (ARIA-H), and have been associated with some fatalities in clinical trials. ARIA are considered an inflammatory reaction to cerebral amyloid angiopathy. Specifically, ARIA-E resembles cerebral amyloid angiopathy-related inflammation, a rare and serious condition caused by auto-antibodies to Aβ. Anti-Aβ antibodies may bind to vascular amyloid, triggering the complement cascade to attack cerebral blood vessels. This process can create small holes, leading to fluid leaks and microbleeds. It is recommended to identify preexisting medical disorders that may predispose individuals to ARIA or increase the likelihood of ARIA-related complications. Such conditions include pre-existing autoimmune or inflammatory disorders, seizures, transient ischemic attacks, cerebrovascular disease, or significant changes in brain white matter.

AD is frequently associated with cerebrovascular disorders, which may contribute to neuronal dysfunction and death. Notably, both conditions share common risk factors, including *APOE ε4*, hyperlipidemia, and obesity [155]. Several lines of evidence support the role of neuroinflammation and cerebrovascular dysfunction in AD. A study involving 508 CU older individuals and 313 patients with MCI and AD found that CSF levels of five biomarkers of neuroinflammation and cerebrovascular dysfunction (YKL-40, ICAM-1, VCAM-1, IL-15, and Flt-1) were elevated in AD, even during the preclinical and prodromal stages, and were associated with CSF tau. Additionally, longitudinal data suggested that higher levels of these neuroinflammatory and cerebrovascular biomarkers were linked to cognitive decline and an increased risk of subsequent development of AD [156].

## Regulatory perspectives on neuroinflammatory biomarkers in Alzheimer’s disease

In the past decade, the identification of biomarkers relevant to AD has become a crucial tool in the development of disease-modifying therapies. Regulatory bodies such as the FDA (https://www.fda.gov/regulatory-information/search-fda-guidance-documents/qualification-process-drug-development-tools-guidance-industry-and-fda-staff) and the European Medicines Agency (EMA) (https://www.ema.europa.eu/en/human-regulatory/research-development/scientific-advice-protocol-assistance/novel-methodologies-biomarkers/opinions-letters-support-qualification-novel-methodologies-medicine-development) have established pathways for the qualification of these biomarkers to facilitate drug development. A qualified biomarker may be defined as a”*tool that, within the stated* *context-of-use, can be relied upon to have a specific interpretation and application in medical product development and regulatory review*” [157]. However, despite the initial EMA opinion on the CSF biomarkers positive signature, which includes low Aβ_1-42_ and high p-tau concentrations, as a predictor for dementia evolution in individuals with MCI (https://www.ema.europa.eu/en/documents/regulatory-procedural-guideline/qualification-opinion-alzheimers-disease-novel-methodologies/biomarkers-use-cerebrospinal-fluid-amyloid-beta-1-42-t-tau-signature/positron-emission-tomography-amyloid-imaging-positive), only a small number of biomarkers have undergone a formal regulatory process for qualification. Significantly, the absence of qualified biomarkers for diagnosing AD, predicting disease prognosis, and evaluating treatment efficacy remains a notable issue. This can be attributed to our limited understanding of the neurobiology of AD and its connection to cognitive and behavioral decline over time. The disease progresses along a continuum of states, which are not fully characterized presently, and exhibits considerable variability among patients. Consequently, the identification and validation of prognostic and predictive biomarkers are urgently required, but their achievement poses substantial challenges.

The FDA recent approval of aducanumab for the treatment of AD through the accelerated approval pathway was met with criticism due to the lack of demonstrated clinical benefit. While the drug induced a reduction in the Aβ biomarker, the EMA did not replicate the FDA approval. The recent FDA full approval of lecanemab, which reduces brain Aβ burden but shows limited cognitive and clinical benefit, further highlights the challenge of using individual biomarkers as efficacy endpoints in AD. The results of recent clinical trials in AD indicate the importance of identifying a broader range of positive biomarker signatures. These should include inflammatory biomarkers, as well as other markers of brain damage or susceptibility to damage. Such biomarkers can be used to monitor and anticipate disease progression across various stages, and to measure the effectiveness of new disease-modifying drugs.

## Discussion

In the AD brain, neuroinflammation is a multifaceted biological process that entails the recruitment of peripheral immune cells, the activation of intracellular signaling pathways, and the release of various proinflammatory cytokines. The key contributors to the neuroinflammatory process are microglia and astrocytes. Their involvement exhibits distinct phenotypic variations, both spatially and temporally, which can be observed at different stages of disease progression [99, 158]. Recent GWAS have provided compelling evidence supporting the significant involvement of the innate immune system and neuroinflammation in the pathogenesis of AD. A comprehensive GWAS conducted on over 1 million participants has specifically highlighted the relevance of microglia and immune cells in the pathogenesis of LOAD [159]. Furthermore, the identification of several AD risk genes associated with immune response and microglia, such as CD33 and TREM2, through GWAS has shed light on their role in the disease [160]. In addition to these genes, sTREM2 and YKL-40, as well as other emerging cytokines, such as IL-6, MCP-1, and TGF-β1 are showing promising potential as inflammatory candidate biomarkers. However, to fully comprehend the clinical implications of these neuroinflammatory biomarkers, it is paramount to conduct large-scale longitudinal studies across the entire AD continuum [13].

sTREM2 has emerged as a promising biomarker of activated microglia and has been validated in longitudinal studies in both pre-clinical and early AD. However, its widespread use is impeded by the fact that it can be easily measured in CSF, but not in blood. Despite this limitation, TREM2 is still considered a promising therapeutic target for AD. One such investigational therapy is AL002, a humanized monoclonal IgG1 antibody that enhances TREM2 signaling to improve microglia survival and proliferation [161]. AL002 is currently undergoing investigation in a 96-week, double-blind, placebo-controlled study that involves 265 patients with early AD (INVOKE-2 study, *ClinicalTrials.gov* NCT04592874). The primary objective of this phase 2 study is to evaluate the impact of AL002 on disease progression, measured through the Clinical Dementia Rating Sum Boxes (CDR-SB).

Numerous cross-sectional and longitudinal studies have consistently shown the potential of YKL-40 as a reliable biomarker of neuroinflammation in AD. YKL-40 serves as an indicator of both activated microglia and reactive astrocytes and can be detected in both CSF and blood samples. Notably, several studies in the field of AD have highlighted the diagnostic significance of plasma YKL-40 levels in the early stages of dementia, such as MCI and mild clinical AD [92, 96, 97]. In addition, elevated plasma concentrations of YKL-40 have been found to be positively associated with cognitive performance in individuals with subjective cognitive complaints [97]. However, it is important to note that increased YKL-40 concentrations in CSF or plasma do not exclusively indicate an inflammatory biomarker specific to AD or other neurodegenerative diseases. Accordingly, elevated YKL-40 concentrations have also been observed in other conditions such as stroke, atrial fibrillation, hypertension, and diabetes, as well as in association with vascular risk factors [86]. The non-specificity of YKL-40 expression in various age-related pathological conditions, including neoplastic and cardiovascular diseases, as well as inflammatory disorders of different etiologies, poses a constraint on its future application as a biomarker in the older adult population [84, 162]. Moreover, while CSF YKL-40 concentrations have shown a moderately positive correlation with p-tau and t-tau, there was no correlation with Aβ, further substantiating its lack of specificity for AD [163]. Therefore, when utilizing YKL-40 in diagnostic examinations, it is crucial to gather a comprehensive medical history of patient comorbidities to avoid misinterpretation of biomarker values [164].

IL-6 shows promise as a potential peripheral inflammatory biomarker for evaluating the severity of cognitive decline. However, there is currently a lack of a standardized molecular panel of fluid inflammatory biomarkers that can be effectively used for screening purposes [33].

**Fig. 3:**
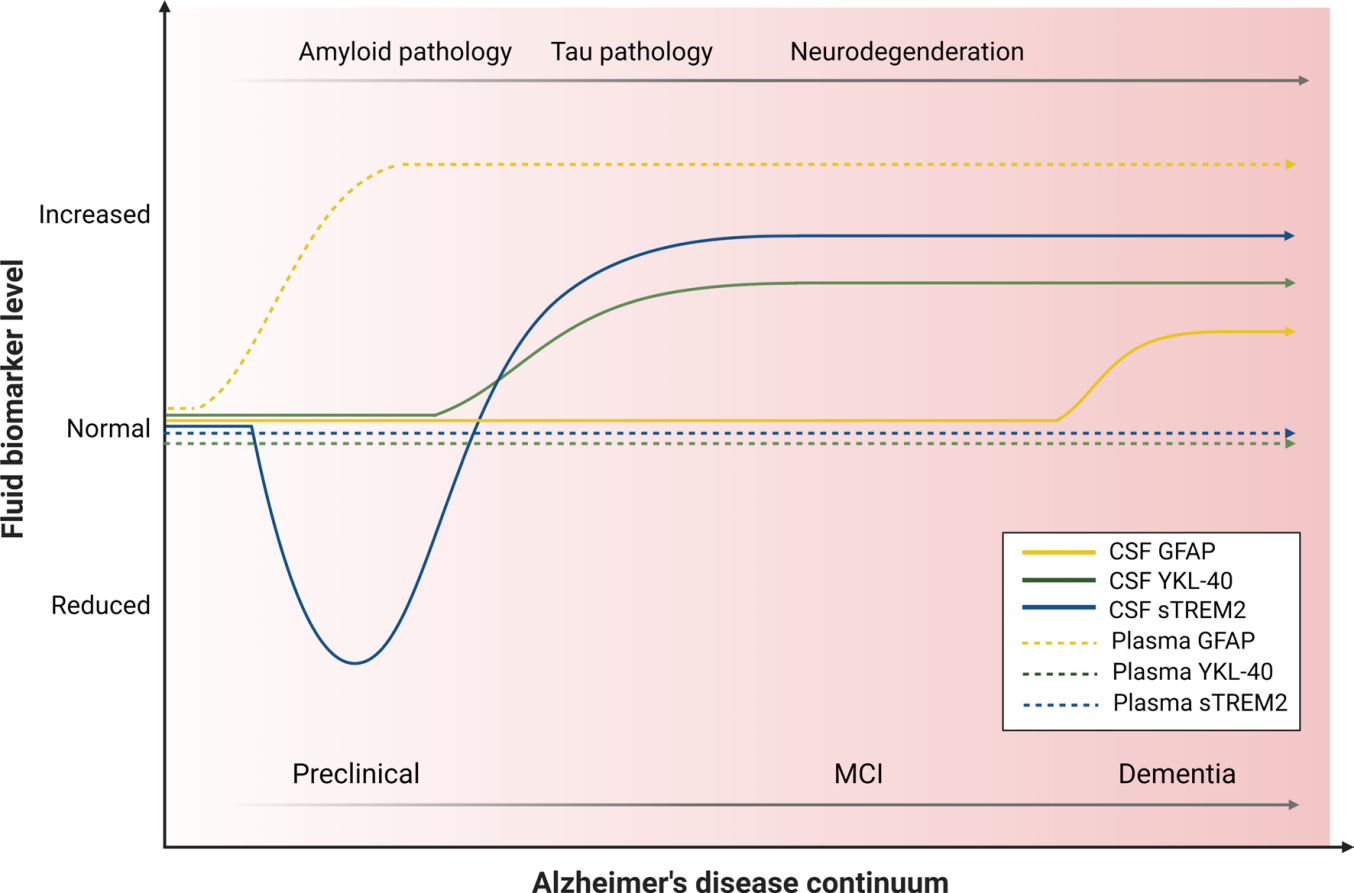
Hypothetical time course profiles of main fluid neuroinflammatory biomarkers (GFAP, sTREM2, and YKL-40) across the AD continuum. The biomarker levels presented in the graph are referred to their normal range and should not be compared with each other. *AD* Alzheimer disease, *CSF* cerebrospinal fluid, *GFAP* glial fibrillary acidic protein, *MCI* mild cognitive impairment, *sTREM2* soluble triggering receptor expressed on myeloid cells 2, *YKL-40* chitinase-3-like protein 1

It is important to note that the literature on inflammatory biomarkers and their ability to track the progression of AD contains some seemingly contradictory findings. A recent 10-year longitudinal study involving CU older individuals identified a positive correlation between CSF sTREM2 levels and the risk of CDR conversion [165]. Conversely, other studies have found that elevated CSF sTREM2 levels are associated with a slower cognitive and clinical decline in AD [166] and in Aβ-PET-positive MCI [167]. Therefore, TREM2-related immune activation may influence the progression of age-related cognitive decline and AD symptoms differently, depending on the disease status and amyloid pathology. It has been hypothesized that throughout the AD continuum, neuroinflammatory biomarkers in blood and CSF exhibit a complex temporal progression, with distinct profiles for CSF and blood sTREM2, GFAP, and YKL-40 [168] (Fig. 3).

This complexity accounts for the apparent discrepancies in the results of some neuroinflammatory biomarker studies and may also partially explain the failure of anti-inflammatory therapies across the AD continuum to date. It is reasonable to assume that anti-inflammatory drugs were tested without considering the inflammatory status of the trial participants. This situation is analogous to the initial studies with anti-Aβ drugs, which were tested in AD patients without assessing their brain Aβ deposition and tau load status. Ideally, future anti-inflammatory candidates should be tested in homogeneous subject populations, characterized by reactive astrocytosis (estimated via plasma GFAP levels) and microglial activation (estimated via CSF sTREM2 levels).

A crucial consideration when utilizing AD neuroinflammatory biomarkers is their lack of specificity. For instance, it is well-established that blood GFAP elevations are not exclusive to AD, as they are also observed in other acute CNS conditions such as ischemic stroke or traumatic brain injury [169]. Similarly, while low plasma sTREM2 has been associated with Aβ accumulation and CSF p-tau levels, a comparable decrease has been reported in vascular dementia [170]. To enhance specificity, a combination of inflammatory biomarkers might be a viable option [139].

However, we posit that the lack of disease specificity in blood biomarkers should not be viewed as a limitation. Instead, it could serve as a valuable initial screening tool. A positive result for a specific blood biomarker test could be interpreted as a non-specific signal of neuroinflammation, neurodegeneration, or Aβ brain deposition, underscoring the need for additional confirmatory testing and further clinical examinations [171].

Currently, the AT(N) classification system is extensively utilized as a biological staging model for AD. It assesses three specific classes of biomarkers, i.e., Aβ, tau pathology, and neurodegeneration/neuronal injury. Recent advancements have identified promising blood-based biomarkers for each category—including Aβ_1-42_/Aβ_1–40_ ratio, phosphorylated tau, and NfL. The Alzheimer’s Association has published recommendations for blood-based biomarkers, emphasizing the need for longitudinal and observational clinical trials to establish changes in peripheral biomarkers over time in patients with AD. These trials should also monitor changes in clinically relevant outcomes, such as cognition and motor functions [172, 173]. Recently, there has been a proposal to enhance the AT(N) matrix by introducing the ATI(N) system, with the addition of a neuroinflammatory biomarker denoted as “I”. The Alzheimer’s Association workgroup is proposing the inclusion of glial GFAP as a biomarker of inflammation and astrocyte activation in their revised diagnostic criteria for AD. This proposal is based on the growing evidence supporting the role of astrocyte reactivity in the pathogenesis of AD. In the near future, it is anticipated that specific “threshold” serum GFAP levels will be established to define “astrogliosis positivity” along the AD continuum [171], providing a standardized approach for assessing astrocyte activation in AD patients. To support this endeavor, a study involving 371 healthy Danish volunteers aged between 21 and 90 years has already determined the normal range of serum GFAP levels across different age groups [174]. This expanded plasma ATI(N) system, in combination with *APOE* genotyping and cognitive testing, presents an opportunity for personalized assessment, enabling a therapy approach tailored to the specific biomarker profiles of patients with AD.

## Conclusion and future directions

Recent longitudinal studies have successfully recruited large cohorts of individuals with accurate clinical characterizations, leading to the identification of potentially reliable blood-based candidate biomarkers of neuroinflammation in AD. These markers offer a practical and non-invasive means of screening and monitoring the inflammatory status of the brain in the AD continuum and hold potential to identify and categorize patients at risk of developing AD, thereby enhancing the screening procedures for potential participants in AD clinical trials and choosing a disease-modifying therapy based on altered biomarker profiles [19].

Longitudinal studies suggest that CSF sTREM-2 exhibits a dynamic response linked to microglial activity as the disease progresses and increased CSF sTREM-2 concentrations are associated with high levels of NfL, indicating axonal injury [76]. CSF sTREM-2 might be validated as a new biomarker for tracking the progression from preclinical AD/MCI to AD dementia that correlates with the progression of CSF Aβ and tau. However, the widespread use of sTREM-2 is impeded by the fact that it can be easily measured in CSF, but not in blood. Despite this limitation, TREM-2 is still considered a promising therapeutic target for AD. One such investigational therapy is AL002, a humanized monoclonal IgG1 antibody that enhances TREM-2 signaling to improve microglia survival and proliferation [161]. AL002 is currently undergoing investigation in a 96-week, double-blind, placebo-controlled study that involves 265 patients with early AD (INVOKE-2 study, *ClinicalTrials.gov* NCT04592874). The primary objective of this phase 2 study is to evaluate the impact of AL002 on disease progression, measured through the Clinical Dementia Rating Sum Boxes (CDR-SB).

According to cross-sectional and longitudinal studies YKL-40 possesses a good potential as a reliable biomarker of neuroinflammation in AD, because its levels are increased in preclinical AD [92] and linked to biomarkers of neurodegeneration (total tau, t-tau), tau-mediated toxicity (p-tau) [89]. YKL-40 might be helpful in distinguishing individuals with MCI who will convert to AD from those who will remain stable at 5 years [86]. Additionally, serum concentrations of YKL-40 can effectively distinguish between CU individuals and those with mild dementia, with a sensitivity and specificity of 85% [164], although it is not clinically useful in differentiating the characteristic AD phenotype and increased YKL-40 concentrations in CSF or plasma have been found in various age-related pathological conditions, posing a constraint on its future application as a biomarker in the older population [84].

When considering all neuroinflammatory biomarkers and all recent longitudinal studies, GFAP emerges as the most promising biomarker, effectively tracking reactive astrocytes and enabling the identification of Aβ-positive CU individuals who exhibit early signs of p-tau pathology, with its plasma concentrations being indicative of early-stage dementia and in MCI who progress into AD [175]. By assessing a combination of plasma biomarkers, such as the ratio between the Aβ_1–42_ and Aβ_1-40_, p-tau_217_, NfL, and GFAP concentrations, it may be possible to create a novel effective panel for assessing the risk of developing AD [19, 116].

According to the evidence discussed in the present review, we believe that enhancing the AT(N) matrix by introducing the ATI(N) system, with the addition of neuroinflammatory biomarkers denoted as “I” (CSF and blood “I” biomarkers), such as GFAP, might significantly improve, in combination with the other biomarkers and cognitive testing, both the early diagnosis of AD and the development of disease-modifying drugs in future AD clinical trials.

## Data Availability

Not applicable.

## Abbreviations

AD: Alzheimer’s disease
ADAD: Autosomal dominant AD
APOE: Apolipoprotein E gene
ARIA: Amyloid-related imaging abnormalities
Aβ: Amyloid-beta
BBB: Blood–brain barrier
bFGF: BBasic fibroblast growth factor
CDR-SB: Clinical Dementia Rating Sum Boxes
CHI3L1 (YKL-40): Chitinase-3-like protein 1
CNS: Central nervous system
CRP: C-reactive protein
CSF: Cerebrospinal fluid
CU: Cognitively unimpaired
FDA: Food and Drug Administration
GFAP: Glial fibrillary acidic protein
GWAS: Genome-wide association studies
hs-CRP: High-sensitivity CRP
IL-15: Interleukin-15
IL-16: Interleukin-16
IL-1β: Interleukin-1beta
IP-10: Interferon-γ-inducible protein 10
LOAD: Late-onset AD
MCI: Mild cognitive impairment
MCP-1: Monocyte chemoattractant protein-1
MMSE: Mini-Mental State Examination
NfL: Neurofilament light chain
NGF: Nerve growth factor
PACC5: Preclinical Alzheimer Cognitive Composite 5
PBMCs: Peripheral blood mononuclear cells
sCD40L: Soluble CD40 ligand
sFLT-1: Soluble fms-like tyrosine kinase-1
sICAM1: Soluble intercellular adhesion molecule-1
SNPs: Single nucleotide polymorphisms
sTREM-2: Soluble triggering receptor expressed on myeloid cells 2
TGF-β1: Transforming growth factor-beta 1
TNF-α: Tumor necrosis factor-alpha
TNFR1: Tumor necrosis factor receptor 1
TNFR2: Tumor necrosis factor receptor 2
TPSO: Translocator protein
VEGF-C: Vascular endothelial growth factor-C
VEGF-D: Vascular endothelial growth factor-D
VILIP-1: Visinin-like protein-1
α1-ACT: α1-Antichymotrypsin
